# Reliable assessment of oral dryness in adults with type 2 diabetes mellitus using the Romanian version of the Summated Xerostomia Inventory

**DOI:** 10.3389/froh.2026.1910215

**Published:** 2026-07-23

**Authors:** Ramona Dumitrescu, Oana Pirvu, Simona Popescu, Simona Olaru-Posiar, Lucian Floare, Vanessa Bolchis, Delia Abrudan-Luca, Doina Chioran, Octavia Balean, Daniela Jumanca, Atena Galuscan

**Affiliations:** 1 Translational and Experimental Clinical Research Centre in Oral Health, Clinic of Preventive, Community Dentistry and Oral Health, University of Medicine and Pharmacy “Victor Babes”, Timisoara, Romania; 2 Clinic of Preventive, Community Dentistry and Oral Health, Department I, University of Medicine and Pharmacy “Victor Babes”, Timisoara, Romania; 3 Doctoral School, “Victor Babes” University of Medicine and Pharmacy, Timisoara, Romania; 4 Second Department of Internal Medicine, “Victor Babes” University of Medicine and Pharmacy, Timisoara, Romania; 5 Department of Diabetes, “Pius Brinzeu” Emergency Hospital, Timisoara, Romania; 6 Faculty of General Medicine, “Victor Babes” University of Medicine and Pharmacy, Timișoara, Romania; 7 Department of Anesthesiology and Oral Surgery, “Victor Babes” University of Medicine and Pharmacy, Timisoara, Romania

**Keywords:** hyposalivation, oral health, patient-reported outcome measures, salivary dysfunction, Summated Xerostomia Inventory, type 2 diabetes mellitus, xerostomia

## Abstract

**Background:**

Xerostomia is a common oral manifestation of type 2 diabetes mellitus (T2DM) and may reflect alterations in salivary gland function associated with chronic metabolic dysregulation. Oral dryness can negatively affect mastication, speech, oral comfort, and quality of life, highlighting the need for reliable patient-reported assessment tools. This study aimed to assess xerostomia in adults with T2DM and to translate, culturally adapt, and validate the Romanian version of the Summated Xerostomia Inventory (SXI).

**Methods:**

A cross-sectional study was conducted in 132 adults with T2DM recruited from a tertiary diabetes outpatient center in Romania. Translation and cross-cultural adaptation followed COSMIN recommendations. Participants completed the Romanian SXI, a global xerostomia question, and a Visual Analog Scale (VAS) for oral dryness. Unstimulated whole salivary flow rate (UWSFR) was additionally measured. Internal consistency, test–retest reliability, concurrent validity, convergent validity, and feasibility were evaluated.

**Results:**

The mean SXI score was 9.58 ± 3.82. Xerogenic medication use was reported by 87.1% of participants, while hyposalivation was identified in 9.1%. Xerostomia-related manifestations included oral burning sensation (21.2%), speech difficulties associated with oral dryness (20.5%), and suspected sleep apnea or habitual snoring (26.5%). The Romanian SXI demonstrated excellent psychometric performance, with high internal consistency (Cronbach's *α* = 0.90), corrected item–total correlations ranging from 0.56 to 0.86, and excellent test–retest reliability (ICC = 0.975). Mean SXI scores increased significantly across xerostomia severity categories (*p* < 0.001), and a very strong positive correlation was observed between SXI and VAS scores (Spearman's *r* = 0.93, *p* < 0.001). No significant floor or ceiling effects were detected.

**Conclusions:**

Xerostomia-related symptoms were common among adults with T2DM, supporting the importance of routine assessment of oral dryness in diabetes care. The Romanian version of the SXI demonstrated excellent validity, reliability, and feasibility and provides a practical instrument for the evaluation and monitoring of xerostomia in Romanian-speaking adults with T2DM. Beyond its application in diabetes care, the Romanian SXI may facilitate the assessment of xerostomia in other populations affected by salivary gland dysfunction, supporting future clinical practice, epidemiological research, and cross-cultural studies in Romanian-speaking populations.

## 1 Introduction

Maintenance of oral homeostasis relies heavily on adequate salivary gland function, and disturbances in salivary secretion can lead to a wide spectrum of oral and functional complications. Reduced salivary flow and xerostomia may impair essential oral functions, including mastication, swallowing, and speech, while also increasing susceptibility to intraoral infections, dental caries, periodontitis, mucositis, oral candidiasis, halitosis, taste alterations, glossitis, and lip fissuring. These clinical manifestations may compromise the use of dental prostheses and contribute to nutritional, behavioral, and social changes (1). Collectively, such consequences have a substantial negative impact on both oral health–related and overall quality of life. Beyond diabetes mellitus, xerostomia is increasingly recognized as a common oral manifestation of several chronic systemic disorders, including chronic kidney disease, autoimmune diseases such as Sjögren's syndrome, and head and neck cancer following radiotherapy. Across these populations, oral dryness has been consistently associated with impaired oral function, reduced oral-health-related quality of life, and an increased burden of oral complications, highlighting the need for standardized approaches to xerostomia assessment (2–4). Nevertheless, diagnosing xerostomia remains challenging due to its inherently subjective nature (5, 6).

Xerostomia is characterized as the subjective perception of oral dryness, with reported prevalence estimates reaching up to approximately 13% in the general population. Its occurrence increases with age, ranging from about 9.3% among individuals aged 15–34 years to nearly 26.5% in those older than 75 years (7, 8).

Type 2 diabetes mellitus (T2DM) is among the most prevalent chronic metabolic disorders worldwide and is associated with a wide range of oral manifestations (9, 10). Xerostomia is one of the most frequently reported oral complaints in individuals with diabetes and may result from multiple mechanisms, including chronic hyperglycemia, autonomic neuropathy, dehydration tendency, polypharmacy, and diabetes-related alterations in salivary gland function (11). Its presence has been associated with impaired oral health, increased caries risk, oral discomfort, and diminished quality of life (12, 13). Consequently, the assessment of xerostomia has become an important component of comprehensive diabetes care and oral health monitoring. Recent studies have emphasized that oral complications of diabetes, including xerostomia, remain underrecognized despite their significant impact on oral-health-related quality of life and diabetes self-management. Accordingly, validated patient-reported outcome measures may improve standardized symptom assessment and support interdisciplinary care (11, 14, 15).

Although salivary gland hypofunction can be objectively quantified through measurements of salivary output and flow rate, the relationship between these parameters and the subjective experience of oral dryness remains weak. Notably, a substantial proportion of patients reporting xerostomia do not exhibit hyposalivation, highlighting the discrepancy between subjective symptoms and objective findings. Consequently, patient-reported outcome measures have been developed to better characterize xerostomia by capturing its multidimensional symptom profile and severity (6, 16).

Previous studies have shown that subjective xerostomia frequently occurs in adults with T2DM even in the absence of objectively measurable hyposalivation, further supporting the need for validated patient-reported outcome measures to accurately assess symptom burden and its impact on daily functioning (11, 13, 17). Because xerostomia is a subjective symptom, its evaluation is best achieved using continuous, multidimensional scales that capture symptom severity along a spectrum rather than relying on binary classification or single-item questions. Patient- reported perception therefore remains central to accurate assessment, requiring instruments that include diverse and discriminative items. When such tools are applied in different linguistic or cultural contexts, cross-cultural validation becomes essential to ensure conceptual equivalence and psychometric validity. This process involves not only linguistic translation but also cultural adaptation in accordance with established international guidelines, so that the instrument retains its reliability and validity within the target population (8, 18).

Among the available instruments, the Xerostomia Inventory (XI), introduced by Thomson and colleagues in 1999, is one of the most widely used multidimensional questionnaires for assessing subjective oral dryness and its functional consequences (5). Originally developed in English for older adults, the XI has subsequently undergone extensive cross-cultural adaptation and validation in numerous languages and populations, including Spanish (2016) (19), Portuguese (2012) (20), German (2019) (21), Korean (2016) (22), French (2021) (8) and Turkish (2022) (6), supporting its broad international applicability. To facilitate its use in clinical and epidemiological settings, van der Putten et al. (23) developed in 2011 a shortened five-item version, known as the Summated Xerostomia Inventory (SXI). Since then, the SXI has been translated and validated in several linguistic and cultural contexts, including Chinese (2013) (24), Portuguese (2017) (1), Indonesian (2019) (25), Hindi (2022) (26),Thai (2024) (27) Given the growing recognition of xerostomia as an important oral manifestation of chronic systemic conditions, and its impact on oral function, quality of life, and long-term disease management, the availability of brief, reliable, and culturally adapted assessment instruments is increasingly relevant in both clinical practice and research. Such instruments may facilitate standardized symptom assessment, support interdisciplinary patient management, and improve the comparability of epidemiological and clinical studies across different populations (11, 17, 23).

Despite the availability of validated versions in several languages, no Romanian version of the SXI has previously been available, limiting standardized assessment of xerostomia in Romanian-speaking populations and restricting participation in international comparative studies. Cross-cultural adaptation of such instruments requires not only accurate linguistic translation but also cultural adaptation to preserve conceptual equivalence, followed by psychometric evaluation to confirm validity and reliability in the target population. Therefore, the aim of the present study was to assess xerostomia in adults with type 2 diabetes mellitus and to translate, culturally adapt, and validate the Romanian version of the Summated Xerostomia Inventory (SXI).

## 2 Materials and methods

### 2.1 Study design and setting

The methodological framework of this study was established in accordance with the COSMIN (COnsensus-based Standards for the selection of health Measurement Instruments) (28) recommendations and internationally recognized guidelines for the cross-cultural adaptation of patient-reported outcome measures (PROMs) (29).

A cross-sectional observational design was employed to evaluate the reliability and validity of the Romanian version of the Summated Xerostomia Inventory (SXI). The study was carried out between January and April 2026 at the Center for Diabetes, Nutrition and Metabolic Diseases of the Pius Brînzeu Emergency Clinical Hospital in Timișoara, Romania.

The study protocol was reviewed and approved by the Research Ethics Committee of the “Victor Babeș” University of Medicine and Pharmacy Timișoara, under protocol number 05/30.01.2024. All participants provided written informed consent prior to enrolment. The study was conducted in full compliance with the ethical principles outlined in the Declaration of Helsinki.

Before initiation of the validation process, the developer of the Xerostomia Inventory, Professor Emeritus William Murray Thomson, was contacted as a professional courtesy and informed about the planned Romanian adaptation. Formal authorization was not required, as the instrument is publicly available. Additional methodological guidance materials provided by the author were consulted to further ensure consistency with current recommendations for xerostomia-related PROM validation studies.

### 2.2 Participants and eligibility criteria

The study population consisted of clinically stable adults with type 2 diabetes mellitus (T2DM) attending routine outpatient follow-up visits at the Center for Diabetes, Nutrition and Metabolic Diseases of the Pius Brînzeu Emergency Clinical Hospital in Timișoara, Romania. Participants were recruited consecutively during the study period, with all eligible patients attending routine outpatient visits being invited to participate until the target sample size was achieved. Of the 140 eligible individuals approached, 8 declined participation, resulting in a participation rate of 94.3%.

Adults with T2DM were selected as the target population because xerostomia and other oral manifestations are frequently encountered in individuals with diabetes mellitus and may be associated with chronic hyperglycemia, autonomic dysfunction, dehydration tendency, altered salivary gland function, and polypharmacy (15, 30). Xerostomia represents one of the most commonly reported oral symptoms in diabetes and may substantially impair oral health, oral comfort, nutritional intake, and oral-health-related quality of life.

Eligible participants were individuals aged 18 years or older with a confirmed diagnosis of T2DM for at least one year. Participants were required to be native Romanian speakers, or sufficiently fluent in Romanian, to ensure accurate comprehension of questionnaire items and response options. Additional inclusion criteria included the ability to independently complete self-administered questionnaires and availability of glycated hemoglobin (HbA1c) measurements obtained within the preceding three months, allowing characterization of current metabolic control.

Exclusion criteria were defined to minimize the influence of conditions independently associated with xerostomia or salivary gland hypofunction. Individuals were excluded if they had a history of head and neck radiotherapy, Sjögren's syndrome or other autoimmune diseases associated with sicca symptoms, severe renal insufficiency or dialysis dependency, active malignant disease undergoing oncologic treatment, acute infection, clinically evident dehydration at the time of assessment, or significant cognitive impairment that could compromise reliable questionnaire completion.

The use of medications with known xerogenic potential, including antidepressants, anticholinergic agents, antihypertensive drugs, and diuretics, was systematically recorded but was not considered an exclusion criterion. This approach was adopted in accordance with COSMIN recommendations in order to preserve external validity and reflect real-world clinical conditions while allowing the potential influence of these variables to be explored during statistical analyses.

### 2.3 Cross-cultural adaptation

The translation and cross-cultural adaptation of the Summated Xerostomia Inventory (SXI) into Romanian were performed in accordance with internationally accepted methodological frameworks for the adaptation of patient-reported outcome measures (PROMs), including the recommendations proposed by Beaton et al. and COSMIN standards (28, 29). The aim of the process was to ensure semantic, conceptual, and cultural equivalence between the original and Romanian versions while preserving the content validity of the instrument.

The adaptation process began with two independent forward translations from English into Romanian. One translation was performed by a clinician with expertise in oral medicine and familiarity with xerostomia-related terminology in order to ensure clinical accuracy. The second translation was carried out by a professional translator without medical background, with the purpose of capturing natural and comprehensible language suitable for the general population. The translators worked independently to maximize identification of ambiguous wording and culturally sensitive expressions.

The two forward translations were subsequently compared and synthesized during a consensus meeting involving the translators and members of the research team. Discrepancies were discussed until agreement was reached, with priority given to clarity, conceptual equivalence, and comprehensibility across different educational levels. This process resulted in a reconciled Romanian version (T12).

Two independent back-translations into English were then performed by bilingual translators blinded to the original questionnaire and without prior familiarity with the instrument. The purpose of this step was to identify inconsistencies, inaccuracies, or potential shifts in meaning introduced during translation.

An expert committee reviewed all versions of the questionnaire, including the original instrument, forward translations, synthesized version, and back-translations. The committee consisted of specialists in oral medicine, a clinician experienced in diabetes care, and a linguist specialized in medical translation. The review process evaluated semantic, idiomatic, experiential, and conceptual equivalence between versions, with particular attention given to cultural relevance and consistency in interpretation of response options.

Minor linguistic refinements were introduced where necessary, resulting in a pre-final Romanian version of the SXI considered linguistically accurate, culturally appropriate, and conceptually consistent with the original instrument. This pre-final version was subsequently subjected to cognitive debriefing in the target population.

### 2.4 Cognitive debriefing (pilot testing)

The pre-final Romanian version of the Summated Xerostomia Inventory (SXI) underwent cognitive debriefing to evaluate its clarity, comprehensibility, and cultural appropriateness in accordance with COSMIN recommendations for PROM validation. Cognitive debriefing was performed in 30 adults with T2DM representing different age groups and educational backgrounds in order to ensure adequate assessment of comprehensibility and cultural relevance.

Participants were initially asked to complete the questionnaire under routine conditions. Subsequently, individual cognitive interviews were conducted by a trained researcher using a structured debriefing guide. The interviews explored participants’ understanding and interpretation of each item, clarity of wording, perceived meaning of the concept of “dry mouth,” linguistic acceptability of the phrasing, ease of selecting response options, and overall relevance of the items to their personal experience.

Particular attention was given to identifying ambiguous wording, misunderstandings, or culturally inappropriate expressions. Signs of hesitation, requests for clarification, and difficulties encountered during questionnaire completion were systematically documented. Overall, participants reported good comprehension of all items and response options, and no major linguistic or conceptual difficulties were identified during the cognitive debriefing process. Consequently, no substantial modifications to the questionnaire were required prior to finalization of the Romanian version. No items were considered confusing, culturally inappropriate, or difficult to understand by more than 10% of participants.

Following this iterative process, the Romanian version of the SXI was considered clear, comprehensible, and culturally appropriate for Romanian-speaking adults. The resulting version was therefore established as the final Romanian version of the Summated Xerostomia Inventory, presented in Table 1.

**Table 1:** Romanian version of the Summated Xerostomia Inventory.

Item	Romanian version
1	Îmi simt gura uscată.
2	Am dificultăți în a mânca alimente uscate.
3	Mi se usucă gura când mănânc.
4	Am dificultăți la înghițirea anumitor alimente.
5	Îmi simt buzele uscate.

Response options for each item were scored on a five-point Likert scale: 1 = “Niciodată”, 2 = “Rareori”, 3 = “Ocazional”, 4 = “Frecvent”, and 5 = “Întotdeauna”. Higher total scores indicate greater xerostomia severity.

### 2.5 Sample size considerations

Sample size was determined in accordance with COSMIN recommendations for studies evaluating the measurement properties of patient-reported outcome measures. COSMIN recommends including at least 5–10 participants per questionnaire item and a minimum sample size of 100 participants to ensure stable estimates of reliability and validity. Given that the Summated Xerostomia Inventory (SXI) comprises five items, a target sample size of at least 100 participants was considered appropriate. A total of 132 participants were ultimately included in the final validation analysis, exceeding the minimum sample size recommended by COSMIN for instruments comprising fewer than 10 items.

For assessment of test–retest reliability, a subsample of clinically stable participants from the main validation cohort was invited to complete the questionnaire a second time after an interval of 7–14 days, in accordance with methodological recommendations for reproducibility studies.

### 2.6 Baseline data collection

Baseline assessments were conducted using a standardized structured questionnaire administered under routine outpatient conditions. Participants initially completed the Romanian version of the Summated Xerostomia Inventory (SXI) as a self-administered mea- sure of perceived oral dryness during the preceding six weeks. The SXI is a shortened version of the original Xerostomia Inventory consisting of five items assessing xerostomia severity. Each item was scored on a five-point Likert scale ranging from “Never” to “Always”, yielding a total score ranging from 5 to 25, with higher scores indicating greater perceived oral dryness. Concurrent validity was assessed using a global xerostomia question (“How often does your mouth feel dry?”) with four ordinal response categories: never, occasionally, frequently, and always. To minimize response bias, the global question was presented separately from the SXI questionnaire.

Convergent validity was further evaluated using a xerostomia-specific Visual Analogue Scale (VAS; 0–10), on which participants rated the overall severity of mouth dryness experienced during the previous six weeks (31).

Demographic and clinical variables were collected to characterize the study population and explore potential associations with xerostomia. These included age, sex, educational level, area of residence, duration of diabetes, most recent glycated hemoglobin (HbA1c) value, diabetes treatment regimen, and presence of diabetes-related complications.

Potential confounding factors known to influence xerostomia were also systematically recorded, including smoking status, alcohol consumption, daily fluid intake, mouth-breathing habits, suspected sleep apnea, and use of medications with xerogenic potential, such as antidepressants, antihypertensive agents, diuretics, and anticholinergic drugs.

Additional oral manifestations and xerostomia-related symptoms commonly reported in adults with T2DM, including oral burning sensation, speech difficulties associated with oral dryness, denture intolerance, mouth breathing, and suspected sleep apnea, were documented for descriptive purposes.

Objective salivary gland function was assessed by measuring unstimulated whole salivary flow rate (UWSFR) using standardized sialometric procedures (32). Participants were instructed to refrain from eating, drinking, smoking, or performing oral hygiene procedures for at least one hour prior to assessment. While seated comfortably, participants were instructed to allow saliva to accumulate in the floor of the mouth and expectorate into sterile 10 mL Eppendorf tubes over a five-minute collection period. Salivary flow rate was subsequently expressed in mL/min. Hyposalivation was defined as an unstimulated whole salivary flow rate ≤0.1 mL/min, in accordance with commonly accepted diagnostic thresholds reported in the literature (33). Measurement of UWSFR was included to explore construct validity, acknowledging that subjective xerostomia and objective salivary flow represent related but distinct constructs that often demonstrate only modest correlation. UWSFR assessment was included to provide an objective indicator of salivary gland function and to explore the relationship between subjective xerostomia and measurable salivary output.

### 2.7 Statistical analysis

Statistical analyses were conducted in accordance with COSMIN recommendations for studies evaluating the measurement properties of patient-reported outcome measures. All analyses were performed using IBM SPSS Statistics for Windows, Version 30.0 (IBM Corp., Armonk, NY, USA). A *p*-value < 0.05 was considered statistically significant. Descriptive analyses were additionally performed to characterize xerostomia-related manifestations, xerogenic medication use, and objective salivary gland function in adults with type 2 diabetes mellitus. Continuous variables were expressed as mean ± standard deviation (SD) for normally distributed data or median and interquartile range (IQR) when distributional assumptions were not met. Categorical variables were presented as frequencies and percentages. Data normality was assessed using the Shapiro–Wilk test.

Feasibility and data quality of the Romanian version of the Summated Xerostomia Inventory (SXI) were evaluated by examining the proportion of missing responses, completion time, and score distribution. No missing data were identified for the SXI questionnaire or for the demographic and clinical variables included in the statistical analyses. Consequently, all analyses were performed using complete-case data, and no imputation procedures were required. Floor and ceiling effects were considered present if more than 15% of participants achieved the lowest or highest possible total score, respectively, in accordance with COSMIN criteria. Internal consistency was assessed using Cronbach's alpha coefficient. Values between 0.70 and 0.95 were considered indicative of acceptable internal consistency. Corrected item–total correlations were additionally calculated, with values ≥0.30 regarded as satisfactory. Test–retest reliability was evaluated in clinically stable participants who completed the questionnaire twice within a 7–14-day interval. The first 40 consecutive participants who agreed to undergo repeat assessment were included in the test–retest analysis. The retest subgroup was drawn from the same validation cohort and fulfilled the same inclusion and exclusion criteria as the overall study population. Before the second administration of the Romanian SXI, participants were asked whether any relevant changes had occurred in their general health status, diabetes treatment, medication use, or oral condition since the initial evaluation. Only participants reporting no clinically relevant changes during the retest interval were considered clinically stable and included in the reproducibility analysis. Stability over time was quantified using the intraclass correlation coefficient (ICC) based on a two-way random-effects model with absolute agreement, in accordance with current recommendations for test–retest reliability studies. ICC values were interpreted as follows: <0.50 poor reliability, 0.50–0.75 moderate reliability, 0.75–0.90 good reliability, and >0.90 excellent reliability.

Construct validity was assessed using hypothesis-testing approaches. Concurrent validity was examined by comparing SXI scores across categories of the global xerostomia question (“never”, “occasionally”, “frequently”, and “always”), with the expectation of progressively increasing SXI scores across severity categories. Between-group differences were analyzed using parametric or non-parametric tests, as appropriate according to data distribution.

Convergent validity was evaluated by assessing correlations between SXI scores and xerostomia Visual Analogue Scale (VAS) scores. Correlation analyses were performed u-sing Pearson's or Spearman's correlation coefficients, depending on data distribution. Correlation coefficients ≥0.40 were considered supportive of convergent validity.

Construct validity in relation to objective salivary gland function was further explored by correlating SXI scores with unstimulated whole salivary flow rate (UWSFR). Based on previous literature, weak-to-moderate negative correlations were anticipated, reflecting the recognized discrepancy between subjective xerostomia and objective salivary secretion. Potential confounding variables, including xerogenic medication use, smoking status, alcohol consumption, and relevant systemic conditions, were explored descriptively. Where appropriate, exploratory sensitivity analyses were performed to evaluate their possible influence on SXI scores.

## 3 Results

### 3.1 Participant characteristics

A total of 132 participants with type 2 diabetes mellitus were included in the final validation analysis. The mean age of the study population was 63.0 ± 10.7 years (range: 32–85 years), and 74 participants (56.1%) were male. Most participants originated from urban areas (67 participants, 50.7%), while 65 participants (49.3%) were from rural environments.

Most participants reported secondary or higher educational attainment. Specifically, 62 participants (47.0%) had secondary education, while 49 participants (37.1%) reported higher or postsecondary education. Primary education was reported by 13 participants (9.8%). Regarding occupational status, retired individuals represented the largest subgroup of the study population (74 participants, 56.1%), followed by professionally active participants (32 participants, 24.2%). Other occupational categories accounted for the remaining participants.

The median duration of diabetes was 5.5 years (IQR: 2–12 years), while the mean glycated haemoglobin (HbA1c) level was 7.06 ± 1.40%. Diabetes-related complications were present in 30 participants (22.7%), predominantly cardiovascular complications and diabetic neuropathy, either isolated or in combination.

Current smoking was reported by 32 participants (24.2%), while 15 participants (11.4%) were former smokers. Occasional alcohol consumption was reported by 55 participants (41.7%). Regarding daily fluid intake, 56 participants (42.4%) reported consuming 1–2 L of fluids per day, 52 participants (39.4%) reported an intake greater than 2 L/day, while 24 participants (18.2%) reported consuming less than 1 L/day.

Medications with xerogenic potential were reported by 115 participants (87.1%). The mean unstimulated whole salivary flow rate (UWSFR) was 0.32 ± 0.15 mL/min, and 12 participants (9.1%) met criteria for hyposalivation.

Additional demographic and clinical characteristics of the study population are summarized in Table 2.

**Table 2:** Demographic, clinical, lifestyle, and salivary characteristics of the study population (*n* = 132). Continuous variables are presented as mean ± standard deviation (SD) or median (interquartile range, IQR), whereas categorical variables are presented as number (percentage).

Variable	Value
Participants, *n*	132
Age, mean ± SD (95% CI) (years)	63.0 ± 10.7 (61.2–64.8)
Male sex, *n* (%)	74 (56.1)
Female sex, *n* (%)	58 (43.9)
Diabetes duration, median (IQR), years	5.5 (2–12)
HbA1c, mean ± SD (95% CI), (%)	7.06 ± 1.40 (6.82–7.30)
Rural residence, *n* (%)	65 (49.3)
Urban residence, *n* (%)	67 (50.7)
Diabetes-related complications, *n* (%)	30 (22.7)
Current smokers, *n* (%)	32 (24.2)
Former smokers, *n* (%)	15 (11.4)
Alcohol consumption (occasional), *n* (%)	55 (41.7)
Xerogenic medication use, *n* (%)	115 (87.1)
Unstimulated salivary flow rate, mean ± SD (95% CI), (mL/min)	0.32 ± 0.15 (0.29–0.35)
Hyposalivation, *n* (%)	12 (9.1)

#### 3.1.1 Xerostomia-related symptoms and salivary dysfunction

Regarding mouth-breathing habits, 27 participants (20.5%) reported predominantly breathing through the mouth, while 46 participants (34.8%) denied this habit. Suspected sleep apnea or habitual snoring was reported by 35 participants (26.5%). Oral burning sensation was reported by 28 participants (21.2%), while speech difficulties related to oral dryness were reported by 27 participants (20.5%). Denture-related difficulties associated with oral dryness were reported by 19 participants (14.4%), whereas 58 participants (43.9%) denied such symptoms. A total of 47 participants (35.6%) reported not wearing removable dental prostheses. Responses were missing or unavailable for 8 participants (6.1%).

**Figure 1:**
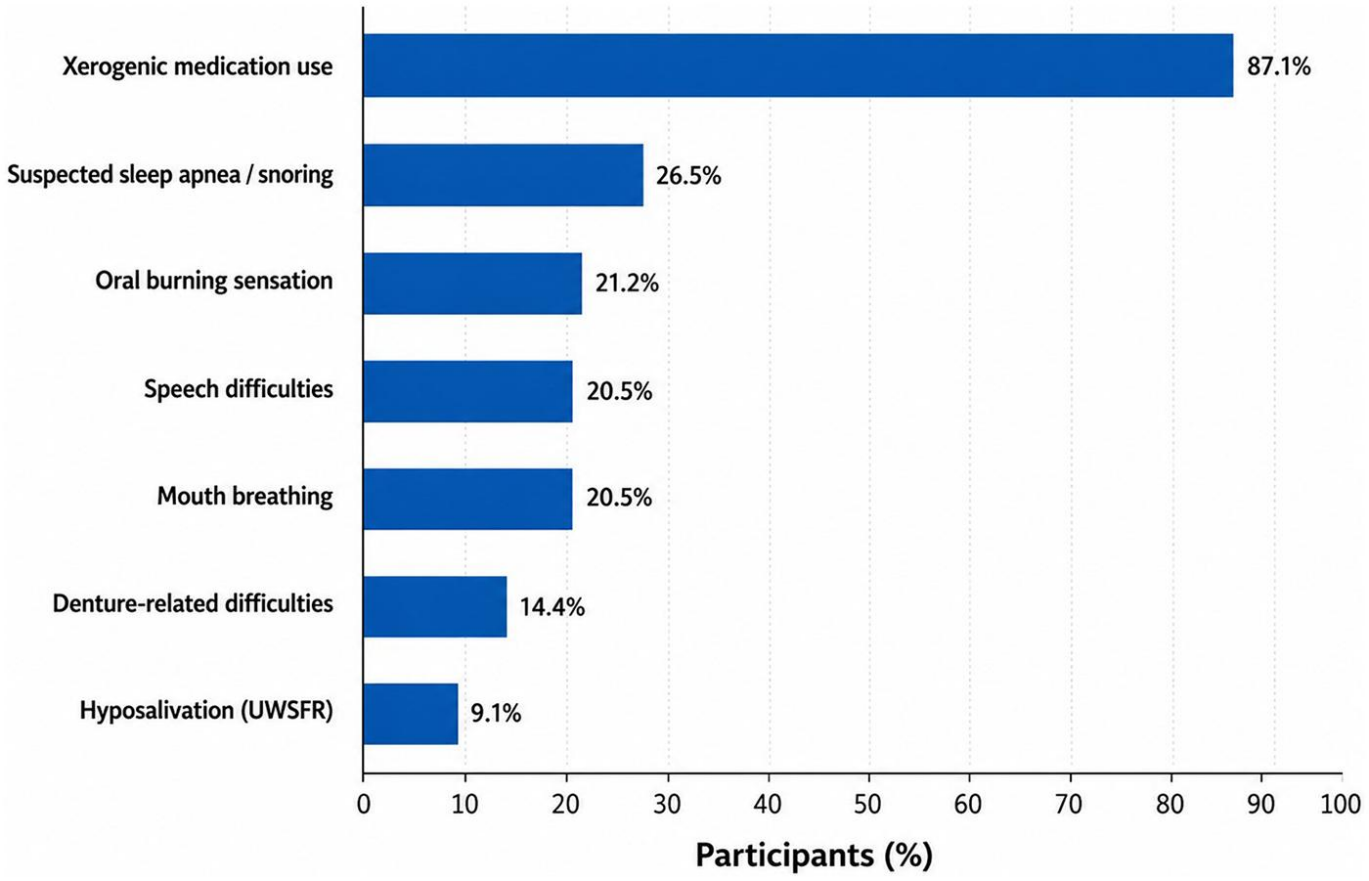
Prevalence of xerostomia-related symptoms, xerogenic medication use, and objective hyposalivation among adults with T2DM.

As shown in Figure 1, xerostomia-related symptoms were relatively common in the study population. Notably, while xerogenic medication use was highly prevalent (87.1%), objective hyposalivation was identified in only 9.1% of participants. This observation further highlights the complex relationship between subjective xerostomia and objectively measurable salivary gland dysfunction.

### 3.2 Descriptive statistics and feasibility of the SXI

All participants successfully completed the Romanian version of the Summated Xerostomia Inventory (SXI), with no missing item-level responses observed. The mean total SXI score was 9.58 ± 3.82, with observed values ranging from 5 to 25, indicating a measurable burden of xerostomia symptoms within this cohort of adults with T2DM. Score distribution demonstrated adequate variability without marked clustering at the lower or upper ends of the scale. No significant floor or ceiling effects were identified. The proportions of participants achieving the minimum and maximum possible scores were 13.6% and 1.5%, respectively, both below the 15% threshold recommended by COSMIN criteria. The questionnaire was generally well accepted and easily completed under routine outpatient conditions, supporting its feasibility in clinical practice.

### 3.3 Reliability

#### 3.3.1 Internal consistency

The Romanian version of the SXI demonstrated excellent internal consistency, with a Cronbach's alpha coefficient of 0.90. Corrected item–total correlations ranged from 0.56 to 0.86, all exceeding the recommended threshold of 0.30 and indicating satisfactory homogeneity among questionnaire items. Detailed item characteristics and internal consistency parameters are presented in Table 3.

**Table 3:** Internal consistency and item characteristics of the Romanian version of the SXI. Corrected item–total correlations are presented for each questionnaire item. Values ≥0.30 were considered indicative of satisfactory item discrimination.

Item	Corrected item–total correlation
Item 1	0.56
Item 2	0.86
Item 3	0.84
Item 4	0.83
Item 5	0.72

#### 3.3.2 Test–retest reliability

Test–retest reliability analysis included the first 40 consecutive participants who agreed to complete the Romanian SXI for a second time within a 7–14-day interval. No participant included in the retest analysis reported clinically relevant changes in general health status, diabetes treatment, medication use, or oral condition between the two assessments. The intraclass correlation coefficient (ICC) for the total SXI score was 0.975(95% CI: 0.954–0.987), indicating excellent temporal stability and reproducibility of the Romanian version of the SXI.

### 3.4 Concurrent validity

**Figure 2:**
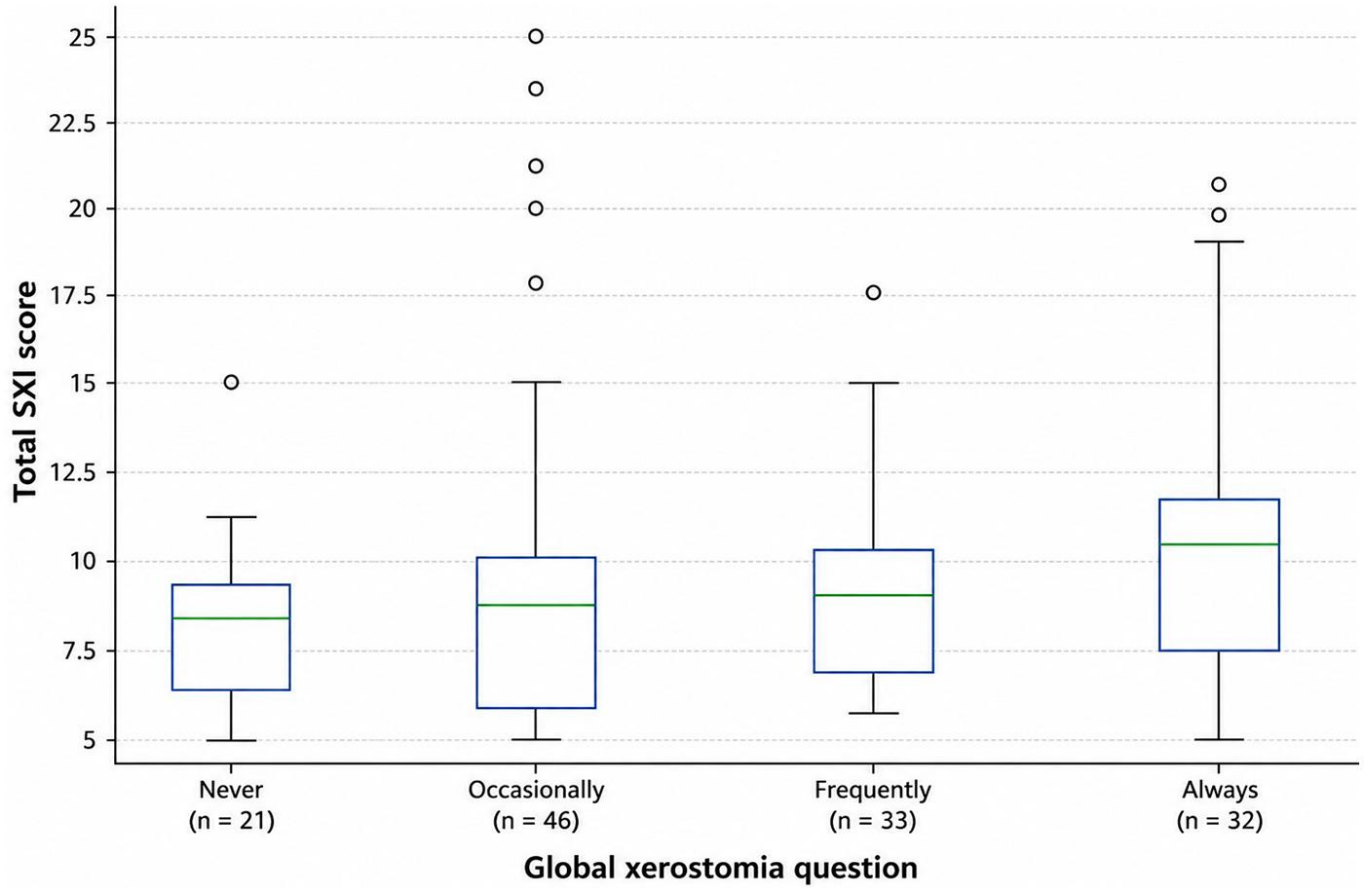
Distribution of SXI scores according to categories of the global xerostomia question.

Mean SXI scores increased progressively across categories of the global xerostomia question, supporting the predefined study hypothesis. Participants reporting “never” experiencing dry mouth demonstrated the lowest mean SXI scores (6.29 ± 2.12), whereas progressively higher scores were observed among participants reporting symptoms “occasionally” (9.56 ± 2.51), “frequently” (13.93 ± 5.51), and “always” (24.00 ± 1.41). Between-group differences were statistically significant (Kruskal–Wallis test, *p* < 0.001), supporting the concurrent validity of the Romanian version of the SXI. The progressive increase in SXI scores across xerostomia severity categories is illustrated in Figure 2.

### 3.5 Convergent validity

SXI scores demonstrated a very strong positive correlation with xerostomia Visual Analogue Scale (VAS) scores (Spearman's *r* = 0.93, *p* < 0.001), supporting convergent validity. The magnitude of this correlation suggests that the Romanian SXI accurately reflects patients’ perceived severity of oral dryness.

### 3.6 Association with salivary flow

Correlation analysis between SXI scores and unstimulated whole salivary flow rate (UWSFR) demonstrated no statistically significant association (Spearman's *r* = 0.01, *p* = 0.887). Although UWSFR represents an objective indicator of salivary gland function, this finding supports the well-recognized discrepancy between subjective xerostomia and measurable salivary secretion.

### 3.7 Clinical and lifestyle factors associated with SXI scores

Exploratory analyses were conducted to assess potential associations between SXI scores and selected clinical and lifestyle-related variables. Participants using medications with xerogenic potential demonstrated slightly higher SXI scores compared with non- users; however, the difference did not reach statistical significance (median SXI score: 10 vs. 8.5, *p* = 0.244). Similarly, no statistically significant associations were observed between SXI scores and smoking status (*p* = 0.070) or alcohol consumption (*p* = 0.508). Female participants demonstrated slightly higher SXI scores compared with male participants (mean ± SD: 10.19 ± 4.22 vs. 9.16 ± 3.69, respectively); however, the difference did not reach statistical significance (Mann–Whitney U test, *p* = 0.106). No significant correlation was observed between SXI scores and glycated hemoglobin (HbA1c) values (Spearman's *r* = 0.09, *p* = 0.311) or diabetes duration (Spearman's *r* = 0.01, *p* = 0.884). Similarly, SXI scores did not differ significantly between participants with and without diabetes-related complications (*p* = 0.469). Given the exploratory nature of these analyses and the primary objective of the present study, the findings should be interpreted cautiously. Because the aim of the study was the cross-cultural adaptation and psychometric validation of the Romanian SXI rather than the identification of independent predictors of xerostomia severity, multivariable regression analyses were not performed.

## 4 Discussion

The present study represents the first cross-cultural adaptation and psychometric validation of the Romanian version of the Summated Xerostomia Inventory (SXI) in adults with type 2 diabetes mellitus. Overall, the Romanian version of the SXI demonstrated very good psychometric performance, including excellent internal consistency, excellent test–retest reliability, satisfactory concurrent and convergent validity, and good feasibility in routine clinical settings. These findings support the use of the Romanian SXI as a reliable and clinically applicable instrument for the assessment of subjective xerostomia in Romanian-speaking adults with T2DM.

The study population consisted of clinically stable adults with T2DM recruited from a tertiary diabetes outpatient center. Xerostomia is one of the most frequently reported oral manifestations of diabetes mellitus and may result from a combination of chronic hyperglycemia, autonomic dysfunction, altered salivary gland function, dehydration tendency, and the widespread use of medications with xerogenic potential. Given the increasing prevalence of diabetes worldwide and the growing recognition of oral manifestations as important components of diabetes care, reliable assessment of xerostomia may contribute to more comprehensive patient management. Recent studies conducted in Romanian adults with T2DM have further highlighted the complex relationships between oral health status, diabetes-related factors, and quality of life, emphasizing the need for comprehensive assessment strategies that include patient-reported oral symptoms and functional impairments (15, 30, 34). Recent international evidence has similarly demonstrated that xerostomia is associated with impaired oral-health-related quality of life, reduced oral function, and greater overall disease burden in adults with T2DM, further supporting the routine use of standardized patient-reported outcome measures in diabetes care (11–13, 35). Moreover, validated PROMs are increasingly recognized as valuable tools for identifying clinically relevant symptoms that may not be fully captured through conventional clinical examination, thereby facilitating earlier intervention and multidisciplinary management involving both dental and endocrinology professionals (36) Consequently, the availability of a culturally adapted and validated Romanian instrument for xerostomia assessment may facilitate both clinical evaluation and future research in this population.

The SXI has previously undergone cross-cultural adaptation and validation in multiple linguistic and clinical settings (6, 8, 19–21). However, previous validation studies of the Xerostomia Inventory and its shortened version have been conducted in heterogeneous populations, including older adults, patients with Sjögren's syndrome, individuals with polypharmacy-related xerostomia, and patients with radiation-induced salivary dysfunction (8, 20, 25). In contrast, the present study specifically focused on adults with T2DM, a population in whom xerostomia represents a frequent yet often underrecognized oral complication. These differences in study populations should be considered when comparing psychometric findings across validation studies. Unlike previous validation studies, which predominantly enrolled older adults, institutionalized individuals, or patients with Sjögren's syndrome, radiation-induced salivary dysfunction, or other causes of chronic hyposalivation, the present study specifically evaluated adults with T2DM recruited from a tertiary diabetes outpatient center. This distinction is clinically relevant because xerostomia in T2DM results from a unique combination of metabolic alterations, autonomic dysfunction, medication use, and diabetes-related complications, rather than from a single underlying condition. Consequently, direct comparisons between language versions should be interpreted cautiously, as differences in participant characteristics, clinical setting, and disease profile may substantially influence both xerostomia severity and psychometric outcomes (8, 11, 19, 20, 25).

The mean total SXI score observed in the present study was 9.58 ± 3.82. This value was lower than that reported in Portuguese (1) and Hindi (26) validation studies, where mean SXI scores ranged between approximately 11 and 12 points, but higher than the value reported in the Indonesian (25) validation study (6.6 ± 2.3). These differences likely reflect variations in study populations, underlying systemic conditions, age distributions, and xerostomia severity. More specifically, the Portuguese validation included individuals with established hyposalivation associated with conditions such as autoimmune diseases, head and neck radiotherapy, and polypharmacy, whereas the Hindi validation was conducted exclusively in patients receiving radiotherapy for head and neck cancer, in whom severe salivary gland dysfunction is expected. By contrast, the Indonesian validation involved older adults residing in nursing homes rather than patients selected according to xerostomia-related disorders. These differences in underlying systemic conditions, age distribution, and baseline severity of salivary dysfunction are likely to have influenced the reported SXI scores across studies (1, 25, 26). In addition, the present cohort consisted exclusively of adults with T2DM, in whom xerostomia may be influenced not only by age and comorbidity burden but also by glycemic status, diabetes duration, medication use, and diabetes-related complications. Furthermore, differences in healthcare organization, access to preventive dental care, and routine integration of oral health assessment into diabetes management may also contribute to variations in symptom recognition and patient-reported xerostomia across countries (11–13, 15, 30). In the present cohort, objective hyposalivation was identified in only 9.1% of participants, suggesting that subjective oral dryness may occur even in the absence of markedly reduced salivary flow. This finding is consistent with the multifactorial nature of xerostomia and may reflect the combined influence of diabetes-related factors, medication use, oral health status, and individual symptom perception.

No significant floor or ceiling effects were identified, with both proportions remaining below the 15% threshold recommended by COSMIN criteria. These findings indicate adequate score distribution and suggest that the Romanian SXI is capable of discriminating between different levels of xerostomia severity without marked clustering at the extremes of the scale. Feasibility was further supported by the absence of missing item-level responses and the good acceptability of the questionnaire under routine outpatient conditions, highlighting its potential utility in both clinical practice and epidemiological research. Comparable findings have been reported in previous validation studies of the SXI, in which floor and ceiling effects were similarly absent or remained below the thresholds recommended by COSMIN, indicating adequate score distribution across different linguistic and cultural settings (1, 25, 26). Likewise, the absence of missing responses and the good acceptability observed in the present study are consistent with previous reports supporting the feasibility of the SXI as a brief patient-reported outcome measure suitable for routine clinical use.

Internal consistency analysis demonstrated excellent homogeneity among questionnaire items, with a Cronbach's alpha coefficient of 0.90. The Cronbach's alpha coefficient observed in the Romanian version falls within the range reported for previously validated versions of the SXI, including the Chinese(*α* = 0.79) (24), Portuguese(*α* = 0.84) (1), Indonesian(*α* = 0.85) (25), and Hindi speaking (*α* = 0.81) (26) populations. In the present study, corrected item–total correlations ranged from 0.56 to 0.86, exceeding the recommended threshold of 0.30 and confirming that all items contributed meaningfully to the assessment of subjective oral dryness. These corrected item–total correlations indicate that each questionnaire item contributed consistently to the overall construct of subjective xerostomia, further supporting the internal coherence of the Romanian SXI. Collectively, these findings support the robustness of the Romanian SXI as a concise and internally consistent patient-reported measure of xerostomia. Moreover, the overall pattern of psychometric performance observed in the Romanian version closely parallels that reported in previous cross-cultural adaptations, further supporting the stability of the SXI across different linguistic and cultural settings.

An important strength of the present study is the assessment of temporal reproducibility through test–retest reliability analysis. The Romanian version of the SXI demonstrated excellent temporal stability, with an ICC value of 0.975 obtained in clinically stable participants reassessed after a 7–14-day interval. This value is comparable to, and in some cases slightly higher than, those reported in previous Portuguese, Indonesian, French, and Chinese validation studies (1, 8, 24, 25), further supporting the high reproducibility of the Romanian SXI over time. The selected retest interval was considered appropriate to minimize recall bias while reducing the likelihood of clinically relevant symptom changes between assessments. Overall, ICC values reported across previous validation studies have ranged from approximately 0.83–0.99, indicating consistently excellent temporal stability of the SXI irrespective of language, cultural setting, or study population. The Romanian version falls well within this range, further supporting its reproducibility.

Concurrent validity was strongly supported by the progressive increase in SXI scores across categories of the global xerostomia question. Participants reporting more severe xerostomia symptoms consistently demonstrated higher SXI scores, supporting the predefined study hypothesis and confirming that the Romanian SXI appropriately reflects subjective xerostomia severity.

Convergent validity analysis further demonstrated a very strong positive correlation between SXI scores and xerostomia VAS scores (Spearman's *r* = 0.93), indicating substantial agreement between two independent patient-reported measures of oral dryness. This finding supports the ability of the Romanian SXI to accurately capture patients’ perception of xerostomia and its severity. The observed correlation was comparable to, or stronger than, those reported in previous validation studies conducted in other linguistic and cultural settings (20, 25). Previous validation studies have similarly demonstrated moderate-to-strong associations between SXI scores and external measures of xerostomia, confirming that the instrument consistently captures patients’ subjective perception of oral dryness across different populations.

The association between subjective xerostomia and objective salivary gland function remains complex and incompletely understood. In the present study, no statistically significant correlation was identified between SXI scores and UWSFR. This finding is consistent with current evidence suggesting that xerostomia and salivary gland hypofunction represent related but distinct clinical entities. Subjective oral dryness may not correlate strongly with salivary flow measurements because symptom perception is influenced not only by salivary quantity but also by salivary composition, oral mucosal sensitivity, neuropathic mechanisms, psychological factors, and individual adaptive responses (37–39). Although UWSFR provides an objective estimate of salivary gland output, it does not reflect qualitative alterations in saliva that are essential for oral lubrication and comfort. Changes in mucin content, salivary proteins, electrolyte composition, buffering capacity, and rheological properties may impair the protective functions of saliva despite preserved secretion rates, thereby contributing to the perception of oral dryness in the absence of measurable hyposalivation (11, 13). Similar observations have been reported in previous validation studies, including the French and Spanish versions of the Xerostomia Inventory, which demonstrated weak or absent associations between subjective xerostomia measures and objective salivary parameters (8, 19).

Interestingly, no significant associations were identified between SXI scores and HbA1c levels, diabetes duration, or the presence of diabetes-related complications. Although these findings should be interpreted cautiously, they suggest that the perception of oral dryness may not be determined solely by metabolic control or disease duration. In adults with T2DM, xerostomia is likely to result from the interaction of several mechanisms, including diabetes-related autonomic dysfunction affecting salivary gland innervation, peripheral neuropathy altering oral sensory perception, chronic metabolic changes within the salivary glands, and the widespread use of medications with xerogenic potential (11, 40). In addition, changes in oral mucosal sensitivity and impaired salivary film stability may further increase the perception of oral dryness independently of objectively measurable salivary flow. These mechanisms may modify the subjective perception of oral dryness independently of objectively measurable salivary flow, further explaining the limited association observed between patient-reported symptoms and salivary secretion rates. Overall, these findings reinforce the concept that xerostomia is a multifactorial symptom resulting from the interaction of biological, neurological, behavioral, and treatment-related factors, emphasizing the importance of direct patient-reported assessment rather than relying exclusively on clinical or laboratory indicators (13). From a clinical perspective, these observations suggest that relying exclusively on objective salivary flow measurements may underestimate the burden of xerostomia experienced by adults with T2DM. Consequently, incorporating validated patient-reported outcome measures into routine clinical assessment may facilitate earlier recognition of clinically relevant oral symptoms and support more individualized patient management (11, 36). Several xerostomia-related manifestations were also documented in the present cohort. Oral burning sensation, speech difficulties associated with oral dryness, mouth breathing, and suspected sleep apnea or habitual snoring were frequently reported despite the relatively low prevalence of objective hyposalivation. These findings further support the multifactorial nature of xerostomia in adults with T2DM and highlight its potential impact on daily functioning, oral comfort, and oral-health-related quality of life (13, 41). Taken together, these findings support the complementary use of objective salivary flow measurements and validated patient-reported outcome measures such as the Romanian SXI, as neither approach alone is sufficient to fully characterize the clinical burden of xerostomia in individuals with T2DM (42). Accordingly, patient-reported measures and objective salivary flow assessment should be interpreted together to provide a more comprehensive evaluation of xerostomia than either method alone.

The relatively high frequency of mouth breathing, suspected sleep apnea, oral burning sensation, and speech difficulties provides additional clinical context for the study population and is consistent with previous reports describing the multifactorial nature of xerostomia and its association with broader oral symptoms in medically compromised individuals (17, 37). These observations are also consistent with recent Romanian studies demonstrating complex relationships between diabetes mellitus, oral health behaviors, oral symptoms, periodontal health, and oral-health-related quality of life (14, 15, 30, 34). Collectively, these findings provide additional clinical context and reinforce the importance of standardized patient-reported assessment of xerostomia in adults with T2DM.

Previous literature has additionally shown that xerostomia and impaired oral health may contribute to nutritional changes, frailty, reduced oral-health-related quality of life, and impaired social functioning, particularly in ageing populations (43). Previous validation studies (6, 25) have variably reported higher xerostomia scores among women, potentially reflecting sex related differences in salivary gland function, hormonal influences, symptom perception, medication exposure, or healthcare-seeking behavior. Although female participants demonstrated slightly higher SXI scores than males in the present study, the difference did not reach statistical significance.

The availability of a validated Romanian version of the SXI provides clinicians and researchers with a brief and easy-to-administer instrument for the assessment of xerostomia in adults with T2DM. Routine implementation of patient-reported measures such as the SXI may facilitate standardized assessment of patient-reported xerostomia symptoms and could support their incorporation into future clinical practice and research (42, 44). Given the high prevalence of dry mouth symptoms in individuals with type 2 diabetes mellitus and their potential impact on oral health, nutrition, speech, oral comfort, and quality of life routine assessment using a validated patient-reported instrument such as the Romanian SXI may may facilitate standardized symptom assessment and support communication between patients and healthcare professionals (11, 15, 42). Whether its routine implementation translates into improved clinical outcomes should be evaluated in future prospective studies. Recent evidence further suggests that systematic assessment of xerostomia should be considered an integral component of diabetes management, as early recognition of salivary dysfunction may facilitate timely preventive interventions and improve oral health outcomes (11, 13). The Romanian SXI may represent a practical instrument for standardized symptom assessment in outpatient diabetes, endocrinology, oral medicine, and dental settings (35, 42). Recent Romanian studies have highlighted significant associations between oral health status, oral-health-related quality of life, oral health behaviors, periodontal health, and diabetes-related outcomes (14, 15, 30, 34). These observations further support the concept that oral health and diabetes interact through multiple biological and behavioral pathways, reinforcing the need for integrated approaches to patient management and oral health assessment (45). From a practical perspective, the Romanian SXI may support standardized communication between endocrinologists, dentists, periodontists, and oral medicine specialists by providing a common patient-reported assessment tool. Because xerostomia is frequently underreported and may occur even in the absence of objectively measurable hyposalivation, systematic use of the questionnaire during routine outpatient visits may facilitate standardized identification of patient-reported symptoms and help identify individuals who could benefit from further dental assessment. Furthermore, repeated administration of the SXI during follow-up visits may allow clinicians to monitor changes in symptom burden over time, evaluate treatment response, and identify patients requiring additional supportive care. Previous studies have also suggested that the Summated Xerostomia Inventory may have value not only as a psychometric instrument but also as a practical screening tool for clinically relevant salivary dysfunction (42). Building on these findings, the Romanian SXI could be incorporated into routine outpatient endocrinology and dental practice as part of periodic patient assessments. Its systematic use may help identify individuals experiencing persistent oral dryness despite apparently stable metabolic control and facilitate timely referral for comprehensive dental or oral medicine evaluation, thereby supporting earlier diagnosis and management of diabetes-related oral complications. Because the SXI is brief and easy to administer, repeated assessments during follow-up visits may also enable clinicians to monitor changes in symptom burden over time and assess patients’ response to clinical management or supportive interventions.

Importantly, validation of the Romanian SXI should not be regarded solely as a linguistic adaptation, but rather as the establishment of a standardized patient-reported outcome measure that enables consistent assessment of xerostomia across clinical practice, epidemiological studies, and future interventional research.

The validation of culturally adapted patient-reported outcome measures is particularly important in multilingual and heterogeneous healthcare settings, as linguistic equivalence alone does not guarantee conceptual or psychometric equivalence across populations (46). In this context, the Romanian SXI may facilitate standardized xerostomia assessment, epidemiological investigations, and cross-cultural comparisons with international studies. Furthermore, although the present validation focused on adults with T2DM, the Romanian SXI may also prove useful in other populations frequently affected by xerostomia, including older adults, patients receiving polypharmacy, individuals with autoimmune disorders such as Sjögren's syndrome, oncology patients undergoing radiotherapy, and medically compromised populations with salivary gland dysfunction. Its availability may therefore facilitate future clinical practice, epidemiological research, and cross-cultural studies involving xerostomia and salivary gland dysfunction in Romanian-speaking populations.

The present study has several strengths. The methodological framework was developed in accordance with internationally accepted recommendations for PROM translation and validation, including COSMIN standards and established cross-cultural adaptation guidelines. The study additionally included objective salivary flow assessment, convergent and concurrent validity analyses, and reproducibility testing through repeated administration of the questionnaire. Furthermore, the evaluation of xerostomia-related symptoms and objective salivary gland function provided additional clinical context regarding the burden of oral dryness in adults with T2DM. The inclusion of adults with T2DM, a population at increased risk of xerostomia, further strengthens the clinical relevance of the validation study. Beyond providing a validated Romanian-language version of the SXI, the present study contributes to the growing international body of evidence supporting the cross-cultural applicability of standardized patient-reported outcome measures for xerostomia. Cross-cultural validation plays a fundamental role in ensuring conceptual and psychometric equivalence across different languages, cultures, and healthcare systems, thereby enabling reliable international comparisons and collaborative research. By validating the instrument in adults with type 2 diabetes mellitus and establishing its psychometric equivalence with previously validated language versions, the study facilitates international comparisons across different healthcare systems and cultural settings and enables the inclusion of Romanian-speaking populations in future multicenter and multinational clinical studies, epidemiological investigations, and pooled analyses evaluating xerostomia and salivary dysfunction. Accordingly, the principal contribution of the present study lies not only in providing a validated instrument for Romanian-speaking patients, but also in expanding the international evidence supporting the harmonized application of the SXI across diverse linguistic, cultural, and healthcare settings.

Several limitations should also be acknowledged. First, the study was conducted in a single tertiary diabetes center, which may limit the generalizability of findings to other Romanian populations or healthcare settings. Moreover, recruitment from a tertiary referral center may have introduced selection bias, as participants attending specialized diabetes services may differ from those managed in primary care or community settings with regard to disease severity, healthcare utilization, and oral health awareness. Consequently, the psychometric performance observed in the present study may not be fully generalizable to all Romanian adults with T2DM, particularly those managed in primary care, rural healthcare settings, or other regions of the country where demographic characteristics, educational level, disease severity, healthcare access, and oral health awareness may differ. Although no evidence suggests that these factors would fundamentally alter the measurement properties of the Romanian SXI, future multicenter validation studies including more diverse populations would further strengthen the external validity and nationwide applicability of the instrument. Second, although the sample size was adequate for psychometric validation purposes, larger multicenter studies may further strengthen external validity. The inclusion of participants from different geographical regions and healthcare settings would improve the representativeness of future validation studies. Third, responsiveness to clinical change was not assessed; therefore, the ability of the Romanian SXI to detect symptom improvement or deterioration over time remains to be established. The cross-sectional design also precludes causal inferences regarding the relationship between xerostomia and diabetes-related variables. Furthermore, although exploratory analyses were performed to examine associations between SXI scores and selected demographic and clinical variables, the study was not designed or statistically powered to identify independent predictors of xerostomia severity using multivariable regression models. Future multicenter studies involving larger and more heterogeneous populations should further investigate independent predictors of subjective xerostomia after adjustment for potential confounding factors. Furthermore, because participants were evaluated at a single time point, longitudinal changes in xerostomia symptoms and their relationship with diabetes progression or therapeutic interventions could not be examined. Although the test–retest analysis demonstrated excellent reproducibility, it was performed in a relatively small subgroup of participants, and future studies including larger retest samples would further strengthen the evidence regarding temporal stability. Another limitation is that structural validity was not evaluated using confirmatory factor analysis. Although the present study focused on the initial cross-cultural adaptation and psychometric validation of the Romanian SXI, rather than a comprehensive evaluation of all measurement properties, this methodological approach is consistent with most previously published cross-cultural validation studies of the SXI, which similarly focused on the core psychometric properties required for the initial validation of a translated instrument (1, 8, 20, 25). The primary objective of the present study was to assess xerostomia in adults with type 2 diabetes mellitus and to translate, culturally adapt, and psychometrically validate the Romanian version of the SXI, with particular emphasis on internal consistency, test–retest reliability, construct validity, and feasibility, in accordance with the COSMIN framework for the evaluation of patient-reported outcome measures (47, 48). Nevertheless, structural validity represents an important measurement property, and future studies involving larger and more heterogeneous Romanian populations should further investigate the factorial structure of the Romanian SXI using confirmatory factor analysis. In addition, measurement error was not formally evaluated using indices such as the standard error of measurement (SEM) or the smallest detectable change (SDC). Consequently, although the Romanian SXI demonstrated excellent reliability, the precision of individual score estimates and the smallest detectable change remain to be established in future longitudinal studies. Accordingly, the findings of the present study should be interpreted as supporting the initial psychometric validation of the Romanian SXI rather than a complete evaluation of all measurement properties recommended by the COSMIN framework. Finally, although objective salivary flow measurements were included, additional analyses of salivary composition or biomarkers were beyond the scope of the present study and warrant further investigation.

Despite these limitations, the Romanian version of the SXI demonstrated excellent psychometric properties and appears to represent a valuable instrument for the assessment of xerostomia in Romanian-speaking adults with T2DM. The findings should nevertheless be interpreted within the methodological constraints of the present validation study and confirmed in larger multicenter and longitudinal investigations. Given the increasing prevalence of diabetes mellitus and the substantial impact of xerostomia on oral health, nutritional status, oral-health-related quality of life, and daily functioning, the availability of a simple, reliable, and culturally adapted screening instrument may facilitate earlier identification and improved multidisciplinary management of oral dryness in both clinical practice and future research settings. The Romanian SXI provides a standardized patient-reported instrument for the assessment of xerostomia in Romanian-speaking adults with T2DM. Future prospective studies are needed to determine whether its routine implementation contributes to improved clinical management, interdisciplinary care, or patient outcomes.

## 5 Conclusions

Xerostomia-related symptoms were common among adults with type 2 diabetes mellitus, highlighting the relevance of oral dryness assessment in adults with type 2 diabetes mellitus. The Romanian version of the Summated Xerostomia Inventory demonstrated excellent psychometric properties, including high internal consistency, excellent test–retest reliability, and satisfactory concurrent and convergent validity. The instrument was easily completed, showed no significant floor or ceiling effects, and proved feasible for use in routine clinical settings. These findings support the Romanian SXI as a reliable, valid, feasible, and standardized patient-reported outcome measure for the assessment and monitoring of subjective xerostomia in Romanian-speaking adults with type 2 diabetes mellitusThe Romanian SXI may represent a practical and standardized patient-reported instrument for xerostomia assessment in Romanian-speaking adults with T2DM. However, future prospective studies are required to determine whether its routine implementation translates into improved clinical management, interdisciplinary care, or patient outcomes. Beyond its application in Romanian-speaking adults with type 2 diabetes mellitus, the validated Romanian version of the SXI contributes to the international harmonization of xerostomia assessment by extending the portfolio of psychometrically equivalent patient-reported outcome measures available for cross-cultural research. Its availability facilitates the inclusion of Romanian-speaking populations in future multicenter and multinational clinical studies, supports epidemiological investigations and cross-cultural comparisons, and contributes to the generation of internationally comparable patient-reported outcome data in xerostomia research.

## Data Availability

The original contributions presented in the study are included in the article/Supplementary Material, further inquiries can be directed to the corresponding authors.
